# Clinical efficacy of laparoscopic radical hepatectomy and laparotomy for hepatocellular carcinoma and factors of postoperative recurrence

**DOI:** 10.3389/fonc.2023.1116984

**Published:** 2023-03-22

**Authors:** Qing Cao, Liang Yang, Guanbao Zhou, Yue Hu

**Affiliations:** Hepatobiliary and Pancreatic Surgery, The First Hospital of Ningbo City, Ningbo, China

**Keywords:** hepatocellular carcinoma, laparoscopic radical hepatectomy for hepatocellular carcinoma, laparotomy, recurrence, high risk factors

## Abstract

**Objective:**

For exploring the clinical efficacy of laparoscopic radical hepatectomy and laparotomy for hepatocellular carcinoma and analysing related factors of postoperative recurrence.

**Methods:**

Totally 212 patients with hepatocellular carcinoma admitted to our hospital between April 2017 and December 2020 were enrolled, and all of them were followed up after the operation. According to the treatment modes, the patients were assigned to a laparotomy group (n=106) and a laparoscopic group (n=106). Perioperative indicators, haematological examination results, complications and recurrence were compared between the two groups. The recurrence time of hepatocellular carcinoma after the operation was confirmed by imaging examination with definite mass, and logistic multivariate analysis was used for analyzing the risk factors associated with postoperative recurrence

**Results:**

Patients in both groups were comparable in terms of general baseline data. The laparoscopic group experienced longer operation time and shorter incision length, less intraoperative blood loss, early time to have the first off-bed activity and time to eat liquid and shorter hospital stay than the laparotomy group (all P<0.05). Seven days after the operation, the laparoscopic group showed notably lower levels of alanine aminotransferase (ALT), aspartate aminotransferase (AST), total bilirubin (TBIL), tumour necrosis factor-α (TNF-α), interleukin-8 (IL-8), and C-reactive protein (CRP) than the laparotomy group (all P<0.05), and also showed notably higher levels of immunoglobulin A (IgA), IgG and IgM than the laparotomy group (all P<0.05). Additionally, the laparotomy group showed a higher total incidence rate of complications than the laparoscopic group (19.81% vs. 9.43%, P<0.05). During the one-year follow-up, the laparotomy group was not greatly different from the laparoscopic group in recurrence rate (22.64% (24/106) vs. 16.98% (18/106), P>0.05). Multivariate analysis showed that average tumour diameter and microvascular invasion were risk factors for postoperative recurrence (P<0.05).

**Conclusion:**

Laparoscopic radical hepatectomy for hepatocellular carcinoma can reduce the influence on liver function and immune function, with less damage to tissues, and can ameliorate postoperative inflammatory reaction, and promote postoperative recovery of patients as soon as possible. There are many factors influencing the postoperative recurrence of hepatocellular carcinoma, and average tumour diameter and microvascular invasion are the risk factors.

## Introduction

Hepatocellular carcinoma (LC) is a frequently-seen malignant tumour of the digestive tract. With the ageing of the population, the incidence of elderly LC is on the rise, which seriously threatens people’s lives and health (1). At the current stage, China is the country with the highest incidence of LC worldwide. There are nearly 780,000 new cases of LC worldwide every year, and over 50% of new patients are in China (2). Because there are no specific symptoms in the early stage of LC, patients often see a doctor only for the cause of complications such as rupture bleeding for LC or bleeding of the digestive tract. Radical hepatectomy of LC is still the main method to treat LC clinically, which can radically remove the primary tumour lesion to alleviate the high tumour load in patients (3, 4). Laparotomy and laparoscopic radical hepatectomy for LC are the main surgical methods for the clinical treatment of LC, but different surgical methods cause different effects on patients (5). Laparotomy can improve the complete resection rate of tumour lesions, but it is greatly traumatic and causes a strong stress response and serious damage to the immune function of the body. In addition, after laparotomy, the tumour can escape, recur and metastasize (6). Accordingly, for patients with LC, laparotomy is a double-edged sword that causes great trauma to the body while removing the lesion.

Laparoscopic radical hepatectomy for LC is currently a minimally invasive method for the clinical treatment of LC, which can relieve the pain of patients and accelerate their recovery without affecting the clinical treatment effect. During this surgery, the surgical vision can be clearly displayed and the lesion can be accurately located to reduce the damage to the surrounding blood vessels and the amount of blood loss, without peeling off muscle tissue. Especially for lesions with more local blood vessels or those prone to occult bleeding after operation, the effect of laparoscopic hemostasis is more thorough (7). Additionally, with this surgery, the surgical incision is small, which reduces the pain of patients, ensures the tightness of the abdominal cavity, reduces the contact bbetweeneen the abdominal cavity the and external environment, and is thus conducive to reducing infection rate and promoting the recovery of patients’ condition (8). Reportedly, compared with laparotomy, laparoscopic radical hepatectomy for LC has the same radical effect in treating LC, with the advantages of clear vision, small trauma, accurate hemostasis, quick recovery and light pain (9).

Radical hepatectomy for LC is the most effective treatment for LC patients, which can substantially improve their prognosis. Although surgery can remove the tumour lesion, the incidence of tumour recurrence and metastasis after surgery may be high due to incomplete resection. In particular, the early recurrence of some patients with LC seriously compromises the long-term survival of patients after surgery, which has become a major problem for clinicians (10, 11). Therefore, this study compared the clinical efficacy of laparoscopic radical hepatectomy and laparotomy for LC, followed up with the patients after the operation, and analyzed the high-risk factors of recurrence after radical resection of LC, with the goal of providing the basis for clinical treatment and prevention of LC recurrence after operation.

## Data and methods

### Research objects

Totally 212 patients with LC admitted to our hospital between April 2017 and December 2020 were enrolled, and all patients were followed up after the operation. The treatment was selected initially based on the patient’s willingness to undergo surgery and the specific situation. According to the treatment modes, the patients were assigned to a laparotomy group (n=106) and a laparoscopic group (n=106). This study followed the ethical standards of clinical trials and was approved by the Medical Ethics Committee of our hospital (No. 2022RS097) and informed consent was obtained from the patients or their families. The two groups were not greatly different in general baseline data (P>0.05), so they were comparable (**Table 1**).

**Table 1:** Comparison of general baseline data between the two groups.

	Laparotomy group (n=106)	Laparoscopic group (n=106)	*χ^2^*/*t*	*P*-value
Gender [n(%)]			0.076	0.783
Male	56	54		
Female	50	52		
Average age (years, `x ± s)	59.27 ± 5.99	58.99 ± 5.88	0.343	0.732
TNM staging [n (%)]			0.172	0.679
Stage I	46	49		
Stage II	60	57		
Average tumour diameter (cm, `x ± s)	4.16 ± 0.32	4.19 ± 0.37	0.631	0.529
Type [n (%)]			1.552	0.213
Regular hepatectomy	51	42		
Irregular hepatectomy	55	64		
The first hepatic portal control [n(%)]			0.931	0.335
Yes	54	61		
No	52	45		
Child-Pugh grade of liver function [n(%)]			0.119	0.730
Grade A	84	86		
Grade B	22	20		

### Inclusion and exclusion criteria

Inclusion criteria: (1) Patients who met the relevant diagnostic criteria of primary LC, and accepted surgical resection treatment after being confirmed by pathological examination; (2) patients without a history of abdominal surgery; (3) patients without serious lesions of important organs such as heart, lung and kidney; (4) patients with karnofsky performance scale (KPS) score ≥60 points; (5) patients with normal mental health and stable vital signs; (6) patients with complete clinical information.

Exclusion criteria: (1) patients with contraindications to laparotomy or laparoscopic surgery; (2) patients with intrahepatic metastasis or distant metastasis in other parts; (3) patients with other malignant tumours; (4) patients with poor cooperation during treatment; (4) patients receiving neoadjuvant or other treatment in the perioperative period.

### Therapeutic regimen

The surgery of both groups was conducted by a relatively fixed group of doctors in accordance with the principle of radical resection of the tumour, with emphasis on the whole excision of tumour and surrounding tissues and the principle of non-contact operation of tumour and sufficient margin.

The control group was treated with laparotomy. General anaesthesia was conducted with tracheal intubation. The patient was let to take a supine position with lower limbs separated. According to the imaging results, the median incision of the upper abdomen or Benz incision was selected. After entering the abdomen, the ligaments around the liver were fully excised by electro tone. Before the operation, the intraoperative bleeding was estimated according to the tumour size and location, and whether to block the first hepatic portal was accordingly decided. The branches of the portal vein to be resected were blocked and marked after the ischemic line was fully exposed. An electric knife was selected to cut the liver parenchyma along the ischemic line from the superficial to the deep to remove the tumour tissue and part of the surrounding normal liver tissue, and then the severed intrahepatic veins, arteries, and bile ducts were ligated. After the resection, effects were made to stop the bleeding fully, take out the specimen, sew up the liver section, fully flush the wound, place the drainage tube, sew the incision layer by layer, and finally close the abdominal cavity.

The observation group was treated with laparoscopic surgery. The methods of anesthesia and preoperative preparation were the same as those of laparotomy. A 10 mm trocar was punctured under the umbilical cord as an observation hole to establish pneumoperitoneum. With the pressure maintained at about 15 mmHg, the abdominal cavity was explored by laparoscopy. According to the position of the lesion, the position of the main operating hole was determined, and the main and auxiliary operating holes were guided to distribute in a fan shape around the diseased liver lobe. The lesion located in the right liver was given the main operating hole under the xiphoid process, and the auxiliary operating holes were located in the midline of the right axilla and right clavicle. The lesion located in the left liver was given the main operation hole under the costal margin of the left clavicle midline, and the auxiliary operation holes were located under the xiphoid process and the right clavicle midline. According to the size and site of the tumour before operation, the intraoperative bleeding was estimated, and whether to block the first hepatic portal was decided. For multiple tumours confined to one segment or lobe of the liver, regular hepatectomy can be selected: (1) Free the liver: Based on the liver segment or lobe where the tumour was located, the corresponding perihepatic ligament was fully freed with an ultrasonic scalpel. (2) Whether to block the first hepatic portal or not was decided according to the actual situation during the operation, and the first hepatic portal blocking band can be placed in advance according to the intraoperative findings. (3) Sever the liver parenchyma: The liver surface tissue was slowly dissected with an ultrasonic scalpel. The deep tissue of the liver was dissected with an ultrasonic scalpel and LigaSure. The liver surface tissue was incised at the position closest to the main supply vessel to find the main supply vessel of the liver segment and then cut off it after clipping. Relatively larger blood vessels were clipped with titanium clips or biological clips and then severed. (4) Specimen treatment: The resected liver tumour was put into a specimen bag after being taken by extending the surgical incision appropriately. Irregular hepatectomy was mainly used for the resection of tumours at the liver margin. Ultrasound scalpel or LigaSure was used to dissect the liver tissue at a distance greater than 1 cm from the margin of the lesion, without dissecting the vascular structure of the hepatic portal. Small blood vessels and bile ducts can be directly cut off with an ultrasonic scalpel or LigaSure; pipes with a diameter greater than 3 mm can be cut off directly with LigaSure or after being clamped with titanium clips. The liver section was washed repeatedly to ensure that there was no bleeding or bile leakage. If necessary, an absorbable hemostatic sponge was placed, and biogel was sprayed to prevent delayed postoperative bleeding. The abdominal cavity was thoroughly cleaned and a drainage tube was placed. The abdomen was closed after the release of the pneumoperitoneum.

### Outcome measures

(1) Perioperative indicators: The operation time, incision length, intraoperative blood loss, time to have the first off-bed activity, time to take liquid diet, and a hospital stay of the two groups were recorded.
(2) All patients were examined by hematology before and after the operation. Elbow venous blood (4 ml) was acquired before the operation and 7 days after the operation, and the serum was separated after centrifugation and placed in the refrigerator for examination. Liver function indexes, including alanine aminotransferase (ALT), aspartate aminotransferase (AST) and total bilirubin (TBIL) were detected, and immune indexes, including immunoglobulin (IgA, IgG, IgM), were also quantified. Serum inflammatory factors including tumour necrosis factor-α (TNF-α), interleukin -8(IL-8) and C-reactive protein (CRP) were determined.
(3) Complications: During the treatment period, the incidence of postoperative complications such as pulmonary infection, abdominal infection, subphrenic abscess, incision infection and bile leakage were recorded in both groups.
(4) Recurrence: Patients were followed up for 1 year from the date of discharge. Within 1 year after the operation, blood tests and abdominal B-ultrasound were re-examined every 3 months, and an imaging examination was performed every 6 months. Patients suspected of recurrence were further examined by CT or MRI. The recurrence time of LC after the operation was confirmed by imaging examination with definite mass, and the postoperative recurrence was recorded. The follow-up ended in April 2022.
(5) The clinical data of the patients were collected, including gender, age, TNM stage, average tumour diameter, operation mode, liver function grade, perioperative indicators, microvascular invasion and postoperative complications.

### Statistical analyses

This study adopted SPSS 22. 0 statistical software for data analyses, and picture drawing using Graphpad prism 9.0. The counting data (n (%)) were compared using the χ2 test. Measurement data were presented by Mean ± SD, and compared using the independent-samples T test between groups, and the Logistics regression model was used for analyzing factors impacting the recurrence, P<0. 05 indicates a statistical difference.

## Results

### Comparison of related perioperative indicators

The laparoscopic group experienced notably longer operation time and shorter incision length, less intraoperative blood loss, early time to have the first off-bed activity and time to eat liquid and shorter hospital stay than the laparotomy group (all P<0.05, **Table 2**).

**Table 2:** Comparison of related perioperative indexes between the two groups.

Group	Operation time (min,`x ± s)	Incision length (cm,`x ± s)	Intraoperative blood loss (ml,`x ± s)	Time to have the first off-bed activity (d,`x ± s)	Time to eat liquid (d,`x ± s)	Hospital stay (d,`x ± s)
Laparotomy group (n=106)	184.70 ± 42.98	20.73 ± 3.34	259.71 ± 59.89	4.39 ± 0.51	6.68 ± 0.87	15.58 ± 3.85
Laparoscopic group (n=106)	231.54 ± 62.55	6.19 ± 1.07	229.46 ± 51.59	2.94 ± 0.67	4.42 ± 0.58	11.73 ± 2.33
*t*	6.354	42.683	3.940	17.730	22.253	8.808
*P*	<0.001	<0.001	<0.001	<0.001	<0.001	<0.001

### Comparison of liver function indexes

**Figure 1:**
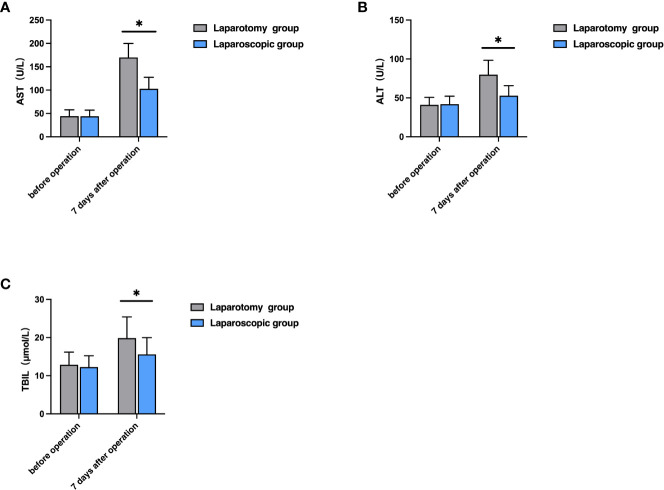
Comparison of liver function indexes between the two groups. **(A)** Comparison of AST level; **(B)** Comparison of ALT level; **(C)** Comparison of TBIL level; *P<0.05).

Before therapy, the two groups were not greatly different in the levels of AST, ALT and TBIL (P>0.05), while seven days after the operation, the laparoscopic group showed notably lower levels of them than the laparotomy group (all P<0.05, **Figure 1**).

### Comparison of immune function indexes

**Figure 2:**
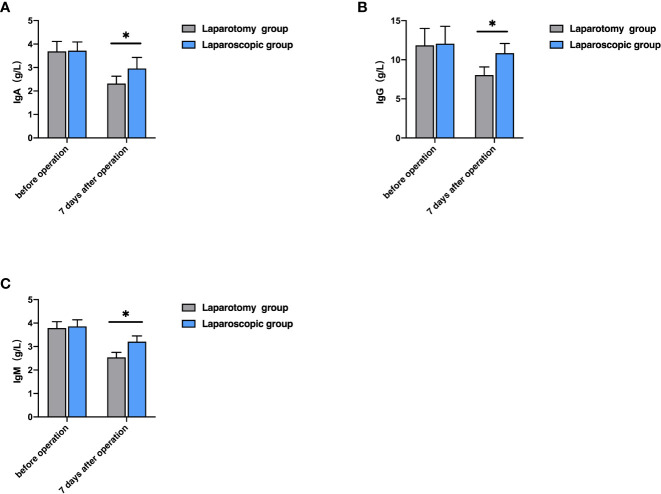
Comparison of immune function indexes between the two groups. **(A)** Comparison of IgA level; **(B)** Comparison of IgG level; **(C)** Comparison of IgM level; *P<0.05).

Before therapy, the levels of IgA, IgG and IgM were not greatly different between the two groups (P>0.05), while seven days after the operation, the laparoscopic group showed notably higher levels of them than the laparotomy group (all P<0.05, **Figure 2**).

### Comparison of serum inflammatory factors

**Figure 3:**
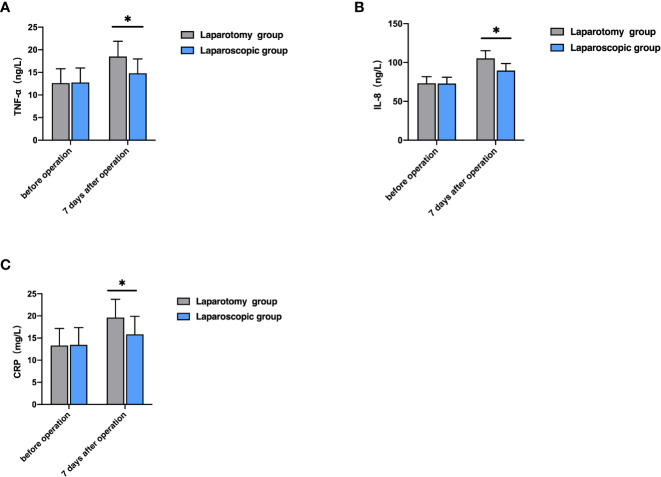
Comparison of serum inflammatory factors between the two groups. **(A)** Comparison of TNF-α level; **(B)** Comparison of IL-8 level; **(C)** Comparison of CRP level; *P<0.05).

Before therapy, the levels of TNF-α, IL-8, and CRP were not greatly different between the two groups (P>0.05), while seven days after the operation, the laparoscopic group showed notably lower levels of them than the laparotomy group (all P<0.05, **Figure 3**).

### Incidence of complications

The laparotomy group showed a total incidence of 19.81%, with 3 cases of pulmonary infection, 4 cases of abdominal infection, 4 cases of subphrenic abscess, 8 cases of incision infection and 2 cases of bile leakage. The laparoscopic group showed a total incidence rate of 9.43%, with 2 cases of pulmonary infection, 3 cases of abdominal infection, 2 cases of subphrenic abscess, 2 cases of incision infection and 1 case of bile leakage. The laparoscopic group showed a notably lower total incidence of complications than the laparotomy group (P<0.05, **Table 3**).

**Table 3:** Comparison of complications between the two groups.

Group	Pulmonary infection	Abdominal infection	Subdiaphragmatic abscess	Incision infection	Bile leakage	Total incidence rate
Laparotomy group (n=106)	3 (41.6)	4 (3.8)	4 (10.4)	8 (2.6)	2 (58.4)	21 (19.81)
Laparoscopic group (n=106)	2 (44.3)	3 (2.5)	2 (11.4)	2 (3.8)	1 (62.0)	10 (9.43)
*χ^2^*	0.205	0.148	0.686	3.778	0.338	4.572
*P*	0.561	0.701	0.407	0.052	0.561	0.033

### Recurrence of LC in postoperative patients

According to one-year follow-up results, as of the end of the follow-up, the laparotomy group was not greatly different than the laparoscopic group in recurrence rate (22.64% (24/106) vs. 16.98% (18/106), χ2 = 1.069, P>0.05).

### The factors impacting the postoperative recurrence

According to univariate analysis with recurrence as the outcome variable, average tumour diameter, microvascular invasion, tumour envelope and portal vein invasion were the factors influencing postoperative recurrence (P<0.05, **Table 4**).

**Table 4:** The related factors of postoperative recurrence.

	Recurrence (n=42)	Non-recurrence (n=170)	*χ^2^*/*t*	*P*
Gender [n(%)]			0.075	0.785
Male	21	89		
Female	21	81		
Average age (years, `x ± s)	58.07 ± 5.53	58.73 ± 5.90	0.657	0.512
TNM staging [n(%)]			0.081	0.776
Stage I	18	77		
Stage II	24	93		
Average tumour diameter (cm, `x ± s)	4.49 ± 0.33	4.10 ± 0.30	7.394	<0.001
Operation mode[n(%)]			1.069	0.301
Laparotomy	24	82		
Laparoscopic surgery	18	88		
The first hepatic portal control [n(%)]			0.073	0.787
Yes	22	93		
No	20	77		
Child-Pugh grade of liver function [n(%)]			1.007	0.316
Grade A	36	134		
Grade B	6	36		
Operation time (min,`x ± s)	222.02 ± 53.44	206.65 ± 57.95	1.562	0.120
Incision length (cm,`x ± s)	13.19 ± 7.29	13.52 ± 7.80	0.249	0.804
Intraoperative blood loss (ml,`x ± s)	242.57 ± 63.04	245.08 ± 56.73	0.251	0.802
Microvascular invasion [n(%)]			24.032	<0.001
Yes	27	41		
No	15	128		
Tumour capsular n(%)]			5.328	0.021
Yes	17	39		
No	25	131		
Portal vein invasion [n(%)]			5.468	0.019
Yes	18	42		
No	24	128		
Postoperative complications [n(%)]			3.541	0.060
Yes	10	21		
No	32	149		

### The risk factors impacting postoperative recurrence of patients

Logistic regression analysis showed that average tumour diameter and microvascular invasion were risk factors for postoperative recurrence (P<0.05, **Table 5**).

**Table 5:** Risk factors impacting postoperative recurrence.

Items	*B*	*S.E*	*Wals*	*P*	*OR*	*95% CI*
Average tumour diameter	1.076	0.293	13.486	0.008	2.933	1.652-5.208
Microvascular invasion	1.284	0.327	15.418	0.003	3.611	1.902-6.855
Tumour capsule	0.762	0.453	2.830	0.529	2.143	0.882-5.206
Portal vein invasion	0.842	0.492	2.936	0.386	2.323	0.886-6.094

## Discussion

With an annually increasing incidence, LC has become a public health problem that seriously compromises residents’ health and quality of life (12). At the current stage, the clinical pathogenesis of LC is still under investigation, but its treatment has captured extensive attention from the medical community. Laparotomy has the advantages of simple operation and clear vision, but it also has the disadvantages of great trauma, great pain and many postoperative complications, which compromise the recovery process of patients (13, 14). Over the past few years, as medical technology advances continuously, the laparoscopic technique gets gradual development clinically and has been extensively used in the field of abdominal surgery, with good therapeutic effects. The application value of laparoscopic radical hepatectomy for LC has also been gradually confirmed (15–17).

In this study, the laparoscopic group experienced longer operation time and shorter incision length, less intraoperative blood loss, early time to have the first off-bed activity and time to eat liquid and shorter hospital stay than the laparotomy group. Laparoscopic radical hepatectomy for LC is at a disadvantage in terms of the operation time, which is not only bound up with the situation during the operation but also closely correlated with the accumulation period of relevant theories and practices in the medical group (18). In laparoscopic surgery, the internal organs are observed in the video, and the operation is completed by endoscopic instruments, with the operation in the abdominal cavity opposite to movement, resulting in the lack of stereoscopic vision and loss of a touch of beginners’ surgical field, which requires high technical requirements for doctors and longer operation time. However, laparoscopic surgery can check the liver and abdominal cavity in multiple directions and angles, without affecting the surrounding organs, and even can display the hidden nerves and blood vessels, which improves the accuracy and precision of the operation, reduces the pain of patients and promotes postoperative recovery (19–22).

Surgery is a kind of traumatic treatment, which can achieve a therapeutic effect, but also leads to a series of physiological changes, affecting the secretion of cytokines and immune function and easily leading to disease recurrence (23). The changes in liver function indexes have certain diagnostic values for treatment effects and disease prognosis. Liver function indexes such as ALT, AST, and TBIL are the main indexes used clinically to judge whether the liver is damaged or not, and their contents in the body can effectively reflect the damage degree. The increase in their contents indicates the aggravation of liver damage (24, 25). IL-8 is produced in monocytes and macrophages, which can chemotactic inflammatory cells to regulate the body’s inflammatory response. However, TNF-α can induce the production of IL-8, and the increase of inflammatory factors such as TNF-α, IL-8 and CRP can aggravate the inflammatory reaction of the body, resulting in local damage to the body (26). Reportedly, laparoscopic radical hepatectomy for LC can alleviate the body’s inflammatory response and promote the body’s postoperative rehabilitation (27). In this study, seven days after the operation, the laparoscopic group showed notably lower levels of ALT, AST, TBIL, TNF-α, IL-8, and CRP than the laparotomy group. The results indicate that laparoscopic radical hepatectomy for LC can reduce the damage to liver function and inflammatory reaction, which is conducive to postoperative rehabilitation by contributing to smaller local body damage. Prior research revealed that both laparotomy and laparoscopic surgery can inhibit the immune function of the body, but laparoscopic surgery causes less damage to the immune function (28). In this study, the laparoscopic group showed notably higher levels of IgA, IgG and IgM than the laparotomy group. Laparoscopic surgery is a minimally invasive operation, with little damage to organism tissues and little influence on immune function, which is beneficial to the rapid recovery of the immune function of patients after the operation.

The trauma of open surgery itself causes the occurrence of postoperative complications, which has taken a crucial negative impact on the clinical outcome of patients with LC after the operation (29). In this study, the results revealed a notably lower total incidence of complications in the laparoscopic group than that in the laparotomy group, which further confirmed that laparoscopic surgery was effective and safe, with the ability to improve the prognosis of patients with LC. In this study, all patients were followed up after the operation. During the follow-up period, the laparotomy group was not greatly different from the laparoscopic group in recurrence rate (22.64% (24/106) vs. 16.98% (18/106). This result suggests that laparoscopic radical hepatectomy for LC is equivalent to laparotomy in the treatment of LC. In the future, the follow-up time can be extended to further explore whether there is any difference in the long-term survival rate and recurrence rate after the operation.

Clinical reports show that postoperative recurrence of LC is influenced by many factors. Tumour diameter is an important influential factor for the postoperative recurrence of LC, which has been recognized by most clinicians. A related study has revealed that a larger diameter of the tumour, especially when the diameter is>5cm, can significantly increase the possibility of tumour recurrence, and its recurrence rate is significantly higher than that of small hepatocellular carcinoma (30). Vascular invasion of hepatocellular carcinoma mainly includes microscopic micro venous invasion and visible large venous invasion, in which microvascular invasion is one of the special intermediate stages in the development of hepatocellular carcinoma. Multivariate analysis in this study showed that average tumour diameter and microvascular invasion were risk factors for postoperative recurrence. The reason may be mainly related to the existence of more blood vessel invasion and more satellite foci besides cancer in hepatocellular carcinoma with larger tumour diameter. Microvascular invasion of hepatocellular carcinoma is mainly manifested by microscopic tiny vein tumour thrombus, and it is an important source and the most direct predictive signal of tumour recurrence with tiny metastasis in the liver, which can lead to occult metastasis and dissemination in the liver after hepatectomy, thus causing tumour recurrence (31).

To sum up, this study has confirmed the efficacy and clinical application value of laparoscopic radical hepatectomy for LC. It can reach the standard of radical resection comparable to laparotomy, with the advantages of small incision, safe and reliable operation, quick postoperative recovery, and good short-term effect. Average tumour diameter and microvascular invasion are risk factors for postoperative recurrence, which provide an important reference for evaluating the prognosis of patients. There is a lack of multi-centre prospective controlled research and clinical observation to verify the long-term curative effect of laparoscopic radical hepatectomy for LC, but laparoscopic radical hepatectomy for LC will demonstrate higher application value after the elimination of disadvantages and expansion of advantages.

## Data availability statement

The raw data supporting the conclusions of this article will be made available by the authors, without undue reservation.

## Ethics statement

The studies involving human participants were reviewed and approved by the Ethics committee of Ningbo First Hospital. The patients/participants provided their written informed consent to participate in this study.

## Author contributions

QC and YH conceived and designed the study and helped to draft the manuscript. LY and GZ performed the data collection and performed statistical analysis. All authors read and critically revised the manuscript for intellectual content and approved the final manuscript. All authors contributed to the article and approved the submitted version.

## References

[1] Orcutt ST Anaya DA. Liver resection and surgical strategies for management of primary liver cancer. Cancer Control (2018) 25(1):621. doi: 10.1177/1073274817744621 PMC5933574 29327594

[2] Zhou M Wang H Zeng X Yin P Zhu J Chen W. Mortality, morbidity, and risk factors in China and its provinces, 1990-2017: a systematic analysis for the global burden of disease study 2017. Lancet (2019) 394(10204):1145–58. doi: 10.1016/S0140-6736(19)30427-1 PMC6891889 31248666

[3] Liu CY Chen KF Chen PJ. Treatment of liver cancer. Cold Spring Harb Perspect Med (2015) 5(9):535. doi: 10.1101/cshperspect.a021535 PMC4561392 26187874

[4] Hu Y Shen ZH. Practice of precision surgery in primary liver cancer. Hepatobiliary Pancreat Dis Int (2021) 20(2):108–9. doi: 10.1016/j.hbpd.2021.01.004 33526404

[5] Wang K Sun JN Wu SD Lu C-D Hu Y-K Ma Y-J. Comparison of the short-term outcomes of open and laparoscopic hepatectomy in the treatment of recurrent hepatocellular carcinoma: a single-center retrospective study. Trans Cancer Res (2022) 11(12):4373. doi: 10.21037/tcr-22-2576 PMC9834575 36644175

[6] Ypsilantis P Lambropoulou M Anagnostopoulos K Kiroplastis K Tepelopoulos G Bangeas P. Gut-barrier disruption after laparoscopic versus open major liver resection in the rat. Surgery (2022) 171(4):973–9. doi: 10.1016/j.surg.2021.11.002 34876288

[7] László P Zoltán M. Laparoszkópia a májsebészetben. MAGYAR ONKOLÓGIA (2018) 62:37–44. 29570185

[8] Schön MR Justinger C. Laparoskopische leberchirurgie [Laparoscopic liver resection]. Chirurg (2017) 88(6):469–75. doi: 10.1007/s00104-017-0413-4 28451728

[9] Sacks GD Lawson EH Tillou A Hines OJ. Morbidity and mortality conference 2.0. Ann Surg (2015) 262(2):228–9. doi: 10.1097/SLA.0000000000001268 26164430

[10] Wang D Zheng X Fu B Nian Z Qian Y Sun R. Hepatectomy promotes recurrence of liver cancer by enhancing IL-11-STAT3 signaling. EBioMedicine (2019) 46:119–32. doi: 10.1016/j.ebiom.2019.07.058 PMC6711863 31375423

[11] Tabrizian P Jibara G Shrager B Schwartz M Roayaie S. Recurrence of hepatocellular cancer after resection: patterns, treatments, and prognosis. Ann Surg (2015) 261(5):947–55. doi: 10.1097/SLA.0000000000000710 25010665

[12] Scherber PR Gäbelein G Eisele RM Igna D Glanemann M. Early stage liver cancer: Hepatocellular carcinoma. Der Chirurg (2018) 89(4):281–8. doi: 10.1007/s00104-017-0538-5 29075797

[13] Zheng M Ma J. Laparoscopic surgery for colorectal cancer with liver metastasis. Zhonghua Wei Chang Wai Ke Za Zhi (2015) 18(6):521–4. doi: 10.1016/j.ebiom.2019.02.017 26108758

[14] Espinel Y Özgür E Calvet L Le Roy B Buc E Bartoli A. Combining visual cues with interactions for 3D-2D registration in liver laparoscopy. Ann BioMed Eng (2020) 48(6):1712–27. doi: 10.1007/s10439-020-02479-z 32112344

[15] Cai XJ Zheng Q Jiang GY. [Current status and prospect of surgical treatment of liver cancer]. Zhonghua Wai Ke Za Zhi (2019) 57(7):494–9. doi: 10.3760/cma.j.issn.0529-5815.2019.07.003 31269609

[16] Peng Y Liu F Wei Y Li B. Outcomes of laparoscopic repeat liver resection for recurrent liver cancer: A system review and meta-analysis. Med (Baltimore) (2019) 98(41):17533. doi: 10.1097/MD.0000000000017533 PMC6799857 31593128

[17] Xu X Guo Y Chen G Li C Wang H Dong G. Laparoscopic resections of colorectal cancer and synchronous liver metastases: a case controlled study. Minim Invasive Ther Allied Technol (2018) 27(4):209–16. doi: 10.1080/13645706.2017.1378236 28925798

[18] Wei Chieh AK Chan A Rotellar F Kim K-H. Laparoscopic major liver resections: Current standards. Int J Surg (2020) 82:169–77. doi: 10.1016/j.ijsu.2020.06.051 32652295

[19] Heinrich S Seehofer D Corvinus F Tripke V Huber T Hüttl F. Vorteile und entwicklungspotenziale der laparoskopischen leberchirurgie [Advantages and future perspectives of laparoscopic liver surgery]. Chirurg (2021) 92(6):542–9. doi: 10.1007/s00104-020-01288-3 32995902

[20] Ciria R Ocaña S Gomez-Luque I Cipriani F Halls M Fretland ÅA. A systematic review and meta-analysis comparing the short- and long-term outcomes for laparoscopic and open liver resections for liver metastases from colorectal cancer. Surg Endosc (2020) 34(1):349–60. doi: 10.1007/s00464-019-06774-2 30989374

[21] Savikko J Vikatmaa L Hiltunen AM Mallat N Tukiainen E Salonen S-M. Enhanced recovery protocol in laparoscopic liver surgery. Surg Endoscopy (2021) 35:1058–66. doi: 10.1016/j.ebiom.2019.02.017\ PMC7886749 32107630

[22] Kirchberg J Reißfelder C Weitz J Koch M. Laparoscopic surgery of liver tumors. Langenbecks Arch Surg (2013) 398(7):931–8. doi: 10.1007/s00423-013-1117-y 24046095

[23] Tang F Tie Y Tu C Wei X. Surgical trauma-induced immunosuppression in cancer: recent advances and the potential therapies. Clin Trans Med (2020) 10(1):199–223. doi: 10.1002/ctm2.24 PMC7240866 32508035

[24] Yu LH Yang Y Zhu B Shen N-J Shi Y Zhao J. Post-operative delayed elevation of ALT and TB> 57.1 umol/L of postoperative day 5 predict posthepatectomy liver failure of patients with HBV-related hepatocellular carcinoma. (2022). doi: 10.21203/rs.3.rs-1893902/v1

[25] Yang HY Jin B Mao YL. Liver injury in COVID-19: What do we know now? Hepatobiliary Pancreatic Dis Int (2020) 19(5):407. doi: 10.1016/j.hbpd.2020.07.009 PMC7377716 32753332

[26] Elsayed DH El-Azzazi FE Mahmoud YK Dessouki SM Ahmed EA. Subclinical endometritis and postpartum ovarian resumption in respect to TNF-α, IL-8 and CRP in Egyptian buffaloes. Anim Reprod (2020) 17(1):e20190027. doi: 10.21451/1984-3143-AR2019-0027 32368278 PMC7190313

[27] Fu XT Tang Z Chen JF Shi Y-H Liu W-R Gao Q. Laparoscopic hepatectomy enhances recovery for small hepatocellular carcinoma with liver cirrhosis by postoperative inflammatory response attenuation: a propensity score matching analysis with a conventional open approach. Surg Endoscopy (2021) 35:910–20. doi: 10.1007/s00464-020-07710-5 32748270

[28] Yang T Lin X Zhang L Yu L Wu Q Zhang S. Integration of IgG and IgA autoantibodies for early diagnosis of hepatocellular carcinoma. Clin Chim Acta (2021) 523:423–9. doi: 10.1016/j.cca.2021.10.037 34728178

[29] Lv X Wu Z Cao J Hu Y Liu K Dai X. A nomogram for predicting the risk of lymph node metastasis in T1–2 non-small-cell lung cancer based on PET/CT and clinical characteristics. Transl Lung Cancer Res (2021) 10(1):430. doi: 10.21037/tlcr-20-1026 33569324 PMC7867781

[30] Nickkholgh A Ghamarnejad O Khajeh E Tinoush P Bruckner T Kulu Y. Outcome after liver resection for primary and recurrent intrahepatic cholangiocarcinoma. BJS Open (2019) 3(6):793–801. doi: 10.1002/bjs5.50217 31832586 PMC6887914

[31] Erstad DJ Tanabe KK. Prognostic and therapeutic implications of microvascular invasion in hepatocellular carcinoma. Ann Surg Oncol (2019) 26:1474–93. doi: 10.1245/s10434-019-07227-9 30788629

